# Machine-learning a virus assembly fitness landscape

**DOI:** 10.1371/journal.pone.0250227

**Published:** 2021-05-05

**Authors:** Pierre-Philippe Dechant, Yang-Hui He

**Affiliations:** 1 School of Science, Technology & Health, York St John University, York, United Kingdom; 2 York Cross-disciplinary Centre for Systems Analysis, University of York, Heslington, United Kingdom; 3 Department of Mathematics, University of York, Heslington, United Kingdom; 4 Department of Mathematics, City, University of London, London, United Kingdom; 5 Merton College, University of Oxford, Oxford, United Kingdom; 6 School of Physics, NanKai University, Tianjin, P.R. China; Fuzhou University, CHINA

## Abstract

Realistic evolutionary fitness landscapes are notoriously difficult to construct. A recent cutting-edge model of virus assembly consists of a dodecahedral capsid with 12 corresponding packaging signals in three affinity bands. This whole genome/phenotype space consisting of 3^12^ genomes has been explored via computationally expensive stochastic assembly models, giving a fitness landscape in terms of the assembly efficiency. Using latest machine-learning techniques by establishing a neural network, we show that the intensive computation can be short-circuited in a matter of minutes to astounding accuracy.

## 1 Introduction

Two facts about simple viruses have been known for a long time. Firstly, that genetic economy leads to the use of symmetry, such that virus capsids are mostly icosahedral or helical. Secondly, packaging signals, that is secondary structure features in the viral RNA, are often required for encapsidation in viruses with single-stranded genomes. Examples are the Origin of Assembly (OAS) sequence in Tobacco Mosaic Virus (TMV), the psi element in HIV (Human Immunodeficiency Virus) and the TR sequence (Translational Repressor) in MS2 (Male Specific 2 bacteriophage). This is an evolutionary advantage, as it ensures vRNA-specific encapsidation and can increase assembly efficiency through a cooperative role of the RNA, which acts as a nucleation site.

**Fig 1:**
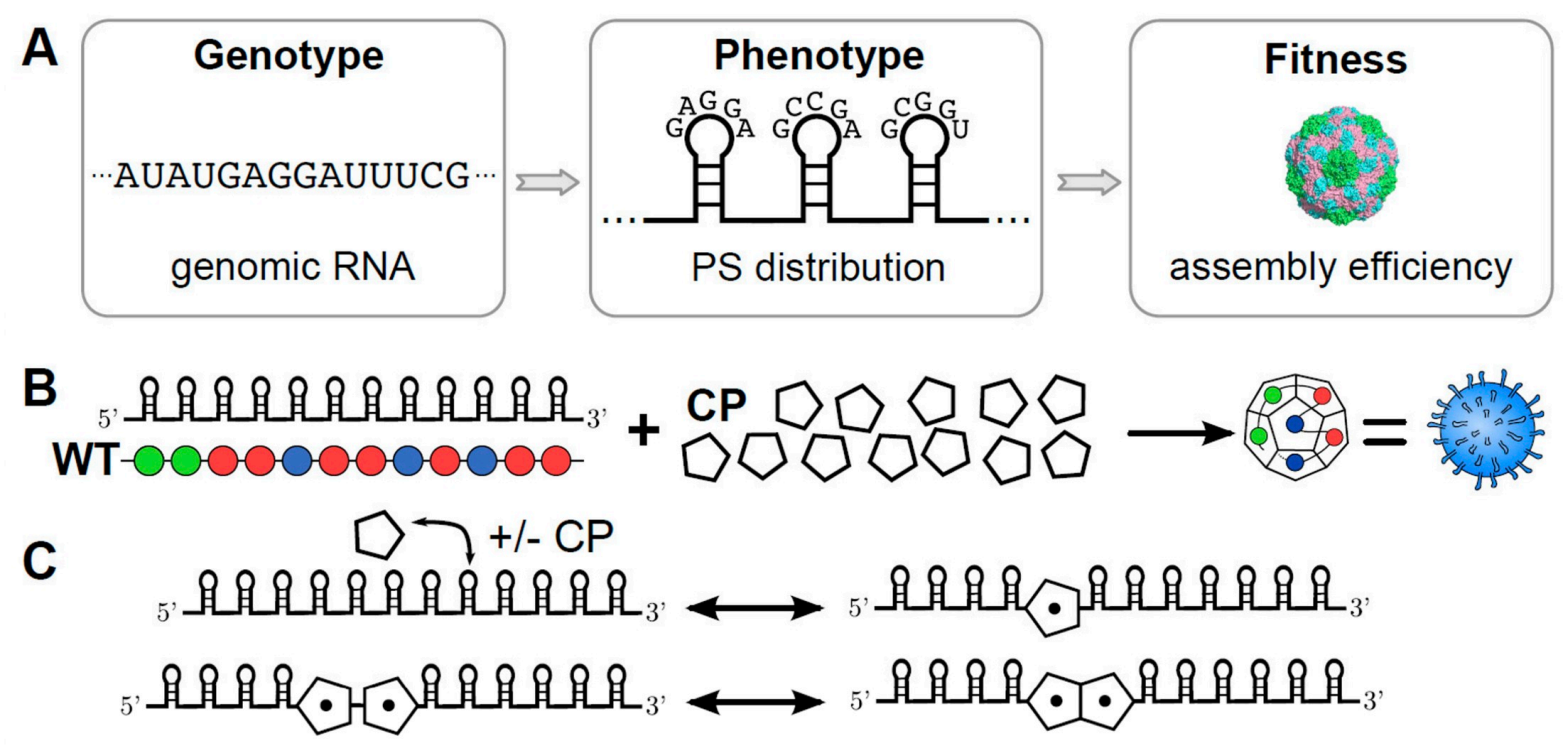
**A** The nucleotide sequence of a virus determines the gene products; however, in addition to this information content the RNA also explores a configuration space of secondary structures. Viruses appear to have evolved to use such motifs to help recruit coat protein with a conserved common recognition motif during assembly. The stability and binding affinity of these packaging signals gives a distinctive profile for viral assembly, which is the phenotype relevant to assembly. Assembly efficiency is the fitness of this phenotype, or at least the contribution to the overall fitness that is determined by aspects of assembly. **B** The genomes in the model consist of twelve packaging signals (PS) that can take weak, medium and strong binding affinities. They successively recruit twelve pentagonal coat proteins, which together form the dodecahedral virion in this model. **C** The stochastic simulation algorithm models several possible reactions. Firstly, packaging signals can bind coat proteins (and fall apart again), and secondly, two coat proteins that have been recruited by packaging signals can bind to each other. The fitness landscape was computed for 2000 virions for each possible genome, making the computations very intensive.

More recently, it has been shown that taken together, these two facts suggest that there could be more than one packaging signal, with multiple signals in fact dispersed throughout the genome [1, 2]. This is because the capsid is symmetric, and the packaging signal mechanism functions via interaction between viral RNA and the coat protein (CP). In several cases, this RNA-CP interaction leads to a conformational change in the CP, which only then makes it assembly competent (e.g. TMV and MS2 [3]. The picture that emerges is then that there are multiple packaging signals (PS) that recruit CP onto a growing capsid. This reduces the phase space that CP has to search in order to assemble a capsid, resulting in vastly increased assembly efficiency. The details of such a mechanism were found in MS2 and STNV (Satellite Tobacco Necrosis Virus) as model systems. Once the details of this mechanism were understood using biochemistry, structural biology [4], bioinformatics [5], biophysics and graph theory [6] in these model systems, related mechanisms could be found in clinically relevant viruses such as Hepatitis C virus (HCV), Hepatitis B virus (HBV) and Human Parechovirus. These packaging signals are secondary structure features of the viral genomes where a stemloop in the single-stranded RNA presents a common recognition motif that can bind to CP (see Fig 1**A**). The viral genome thus has multiple layers of constraints, by having to code for genes as well as the PS instruction manual. This set of packaging signals can also be repurposed and optimised for the assembly of virus-like particles, which do not share the same genetic constraints as the virus, and could be used e.g. for vaccines, drug delivery or as an anti-viral strategy [7].

An equilibrium model of how to assemble a simplified example of an icosahedral virus, a dodecahedron built from 12 pentagonal faces, was considered in [8] using an ODE (ordinary differential equations) model. More recently, the multiple dispersed packaging signal paradigm has sparked renewed interested in such a dodecahedral model [9]. The assembly reaction kinetics was modelled via a set of discrete reactions in a stochastic simulation paradigm based on the Gillespie algorithm [10].

In this model 12 PSs can bind CP, as well as dissociate again, reflecting reversible/equilibrium kinetics. Bound CP can then bind other bound CP, gradually building up a capsid (see Fig 1**B** and 1**C**). The PSs here have three different bands of binding affinity: weak, medium and strong. These choices correspond to binding energies of 4/8/12 kcal/mol respectively, based on the TR sequence in MS2 which has approximately 12kcal/mol. The binding energy between CPs is much lower, at approximately 2 kcal/mol. This modulation of affinity affects the assembly kinetics, e.g. by providing a nucleation point that starts assembly, or allowing for error-correction via weaker binding elsewhere. The thermodynamics of PS binding and of the number of CP bonds formed then translates into assembly efficiency. This in turn is taken as a proxy for fitness (all other things being equal)—or at least the contribution to the fitness that results from assembly considerations [11–13].

In [14] the whole space of these 3^12^ genomes (or rather, phenotype profiles) has been explored. The assembly efficiency there is given by the number of capsids that have correctly assembled out of a possible total of 2, 000. This efficiency provides a fitness landscape on the 12-dimensional genome space. This is an interesting model that is tractable, in contrast with many other biological systems, as it has a small number of degrees of freedom and is dominated by the symmetry of the capsid. This tractability also allows for the consideration of viral evolution. For instance, mutation of the PS strengths leads to the emergence of a set of related genomes that form a ‘quasispecies’ [15, 16]. One can thus investigate the effect of evolutionary pressures, e.g. those exerted by standard drugs or a novel type of drug that targets packaging signals [9].

This model thus captures many interesting aspects of viral genetics, geometry and assembly. A more realistic model would have more CP building blocks and PSs, e.g. around 60 for MS2 (i.e. one full orbit of the icosahedral group). But the computation time for even these simple genomes and the assembly kinetics that provide the fitness landscape are already considerable. Even other simplified models, e.g. reduced orbits on symmetry axes given by e.g. an icosahedron consisting of 20 triangles with 20 PSs, a rhombic triacontahedron consisting of 30 rhombuses with 30 PSs, or a finer gradation of binding affinity bands are already computationally out of reach. For experimental approaches to measure local fitness values please see for instance [17], though note that in this reference this is limited to only 48 data points, whereas the fitness space of the simple above model is already 10; 000 times that.

## 2 Results/Discussion

This data set is a perfect example of data that is amenable to a machine-learning approach, since it associates a vector input with a number output. We therefore train a neural network to predict the fitness landscape. The network is trained on a subset of the whole genome space, and validated on the remainder of the data. This proof-of-principle shows that it is very fast for a neural network to learn the inherent patterns within the large degeneracy of the detailed stochastic modelling to predict assembly efficiency fitness for unseen genomes to extremely high accuracy (c.f. the paper [18] which has been published after submission of this manuscript that applies machine learning to the related problem of finding high-level behaviour within the large degeneracies of protein folding). The danger is that some subtleties of the stochastic modelling are lost, but allowing for computation times many orders of magnitude faster. More likely, however, since the data is obtained from a Monte Carlo simulation, and many works in the literature immediately go to an ODE approximation and miss these details anyway, the ML is actually indifferent to this. In fact, the discontinuities from the stochastic method, which the ODE smoothing would not capture, is perfectly adapted to the neural network and machine learning classifiers, which are much better adapted to partitioning fitness space in more subtle ways. Many different neural network architectures were tested and all led to very similar results. This supports the point that there is something reasonably simple underlying the data set that can be learned by any reasonable neural network. This approach could thus in future be used to tackle more realistic models such as the ones mentioned above. Stochastic simulations could be used to partially explore these larger genome spaces, calculating assembly fitness in order to provide a training set for a neural network. The rest of the fitness landscape can then be predicted by the artificial intelligence; it is also possible to only compute this fitness if necessary, e.g. when a new genome arises through mutation in a quasispecies model, such that such computation may only be necessary ‘precedurally’. By that we mean that they are only calculated locally when required during the computation, e.g. when evolutionary dynamics starts exploring a certain range in fitness space, and not calculating the entire fitness space before beginning the simulation.

The assembly process discussed here is ultimately a problem of geometry and thermodynamics. So it would with some modification also apply to the assembly of other icosahedral particles such as virus-like particles (VLPs) for drug delivery or as vaccines, as well as to carbon fullerene assembly, which are very attractive fields for biomedical and nanoscience applications. For instance, virus-like particles could present viral epitopes in order to act as vaccines. In order to find the most efficient assembly pathway for such VLPs, an analogue of the above fitness landscape could be constructed in order to solve the resulting optimisation problem and to give industry suggestions which parts of the fitness landscape to explore deeper experimentally.

## 3 Methods

From a purely mathematical point of view, we have the following problem. Let (weak, medium, strong) be denoted respectively by (1, 2, 3). The input is a vector *v* in a 12-dimensional vector space over _3_, the field of three elements. The output is an integer (which we treat as a real number) between 0 and 2000, which we can normalise into *ϵ* ∈ [0, 1] ⊂ by dividing by 2000. The algorithm used by [14] is thus a map

$$\begin{array}{c} f\;:\;v\in_{3}^{12}⟶\epsilon \in \left[0,1\right]\;. \end{array}$$ (1)

A typical example is

$$\begin{array}{c} \left\{1,1,1,2,2,2,3,1,2,2,1,1\right\}⟶\frac{1523}{2000}≃0.7615 \end{array}$$ (2)

### 3.1 Computational aspects of the simulation

The map *f* is a computationally intensive one with individual genome run times between 20 minutes and 12 hours, and cumulative run time of 3-4 weeks on the N8 Polaris high performance computing research cluster, Intel 2.6 GHz Sandy Bridge E5-2670 processors, with a total of 5, 312 cores, with a mix of 4 and 16Gb of RAM (https://n8hpc.org.uk/facilities/) [9]. The fluctuations in numbers of assembled capsids tend to be in the tenth of a percent range (i.e. ±20 virions), however when initially running the code with 75 repeats of each run for certain genomes the standard error was very small (±0.001%) [9]. Due the amount of time and cluster resources it already took, each point on the landscape is the result of only 2 simulation runs. While not ideal, cluster computation was limited; the simulation allows to capture the more generic features whilst the above cross-validation suggests that the error is small [9]. Nevertheless a brute-force simulation has been performed on the 3^12^ = 531, 441 possible input values and the efficiency value extracted. This gives us a database of some half a million known cases of the form (2).

### 3.2 Machine-learning the dataset

Such a problem is perfectly adapted to supervised machine-learning: we know many input values and wish to train some artificial intelligence to associate the input with the known output on some small subset, and use it to predict the output for unseen input [19]. The advantage of this approach is that often approximate results can be attained at reduction in computation time by many orders of magnitude. The paradigm of using machine-learning in algebraic geometry and more general classes of problems in pure mathematics was proposed in [20–22] to satisfying accuracy, and it is a similar philosophy that we will adopt here.

**Fig 2:**
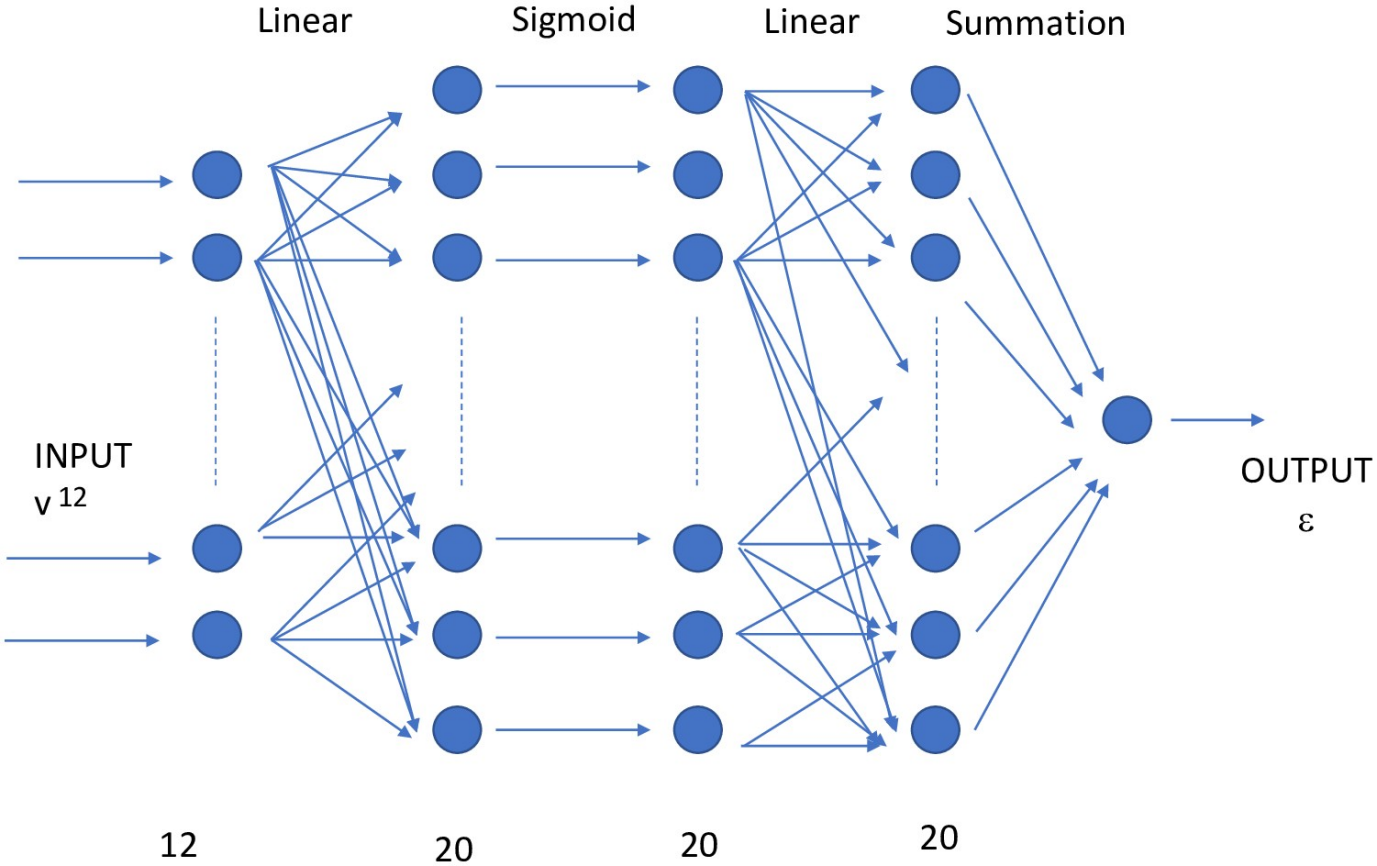
The structure of the neural network used in the calculation.

Let us first try the following specific procedure:

- Take the full data *D* of the form (2), of size 3^12^;
- Establish the neural network, a 3-layer perceptron
  INPUT=v→L20→S20→L20→Σ1→OUTOUT=ϵ
  INPUT=v→L20→S20→L20→Σ1→OUTOUT=ϵ
- In the above, L means a linear-layer, S, a sigmoid layer and Σ, a summation layer. In particular, the first linear layer L_20_ is a fully connected layer taking the 12-vector *v* to 20 neurons by simply the linear function *y* = *wx* + *b*. This is then fed into an element-wise sigmoid layer $\sigma \left(x\right)={\left(1+e^{-x}\right)}^{-1}$ of 20 neurons, followed again by a linear layer, which is then summed to the real number *ϵ* as the output. The schematic of is shown in Fig 2. We have taken this neural network only to illustrate the power of our methodology and have not optimised the hyper-parameters such as 20, nor the network architecture or the choice of the type of neurons.
- Now split *D* into a training set *T* of 30,000 random samples; the validation set will be the complement *V* = *D*\*T*. Note that the training data is only about 5.6% of the total data.
- We train with *T* and validate on *V*.
- As a further check, we create a “fake” validation set $\tilde{V}$ which has the same inputs as that of *V* but with output randomly assigned from the set of correct outputs.

On an ordinary laptop (Intel Core m5-6Y57 CPU, 1.10GHz, 2 Cores, 4 Logical Processors, with 8Gb of RAM), the training took about 45 seconds, and the prediction about 10 seconds. The algorithm is implemented on Mathematica [23] and is expected to run even faster on the Python package Tensorflow [24]. A python Jupyter notebook is attached along with the Mathematica notebook as S1 and S2 Files. In other words, the entire computation took under 1 minute with an ordinary notebook as opposed to the many hours it took on a super-computer. As mentioned above, a number of similar neural networks were all able to perform this supervised learning task in a comparably short time, meaning there was little motivation to optimise neural network architecture or hyperparameters. However, this reinforces the point that there is intrinsic structure in the costly simulation dataset that any reasonable neural networks finds easy to learn. We also tried various combinations of other standard machine-learning algorithms such as decision trees and support vector machines, and empirically find that our particular neural network approach above seems to out-perform all of them.

**Fig 3:**
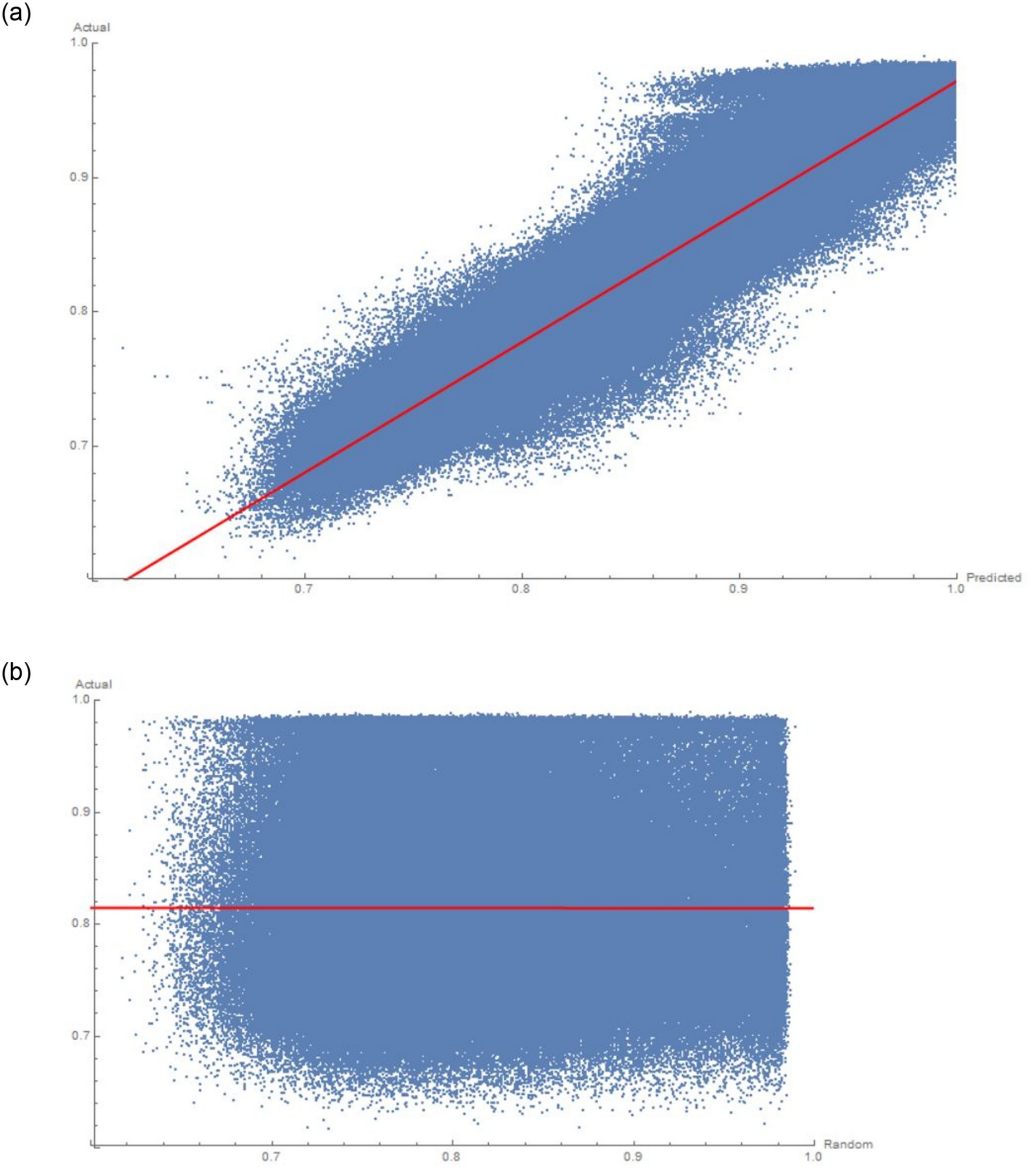
**A** A scatter plot of the predicted and actual value of *ϵ* for the validation data from a 30, 000 training sample; **B** scatter plot of a random-prediction versus the actual *ϵ* value.

We present the result in Fig 3. In part **A**, we present a plot of the predicted *ϵ* on the horizontal versus the actual *ϵ* on the vertical. There are 501, 441 points. One can see that they cluster near the desired *y* = *x* line, which would mean perfect prediction (note the axis ranges). To give some precise measures, the best fit line is *y* = −0.0262122 + 1.02519*x* with F-statistic 2.45082 × 10^6^ and p-value less than $10^{-10^{6}}$. The R-squared value is 0.830151. The errors themselves (fit-residuals) give a mean and standard deviation of (6.88 ± 0.02) × 10^−16^, showing that the residuals are unbiased around 0. To double check, we plot the same result for the fake validation set $\tilde{V}$ in part **B**. It is obvious that the distribution is much less structured and essentially randomly occupies a square. The fit here is *y* = 0.815233 − 0.000947474*x* i.e. practically a constant, with a poor F-statistic of 0.450401 and a poor p-value of 0.502145. The R-squared value is 8.98217 × 10^-7^. This is very re-assuring for less than 6% of seen data and total computation time of less than 1 minute on an ordinary laptop.

To give an idea of the prediction, for the (1, 1, …, 1) vector, the net predicts *ϵ* = 0.87069, or 1741. The original value is 200, but that is a singular outlier in the whole data set, which we would not expect the neural network to be able to reproduce. For the (2, 2, …, 2) vector, the net predicts *ϵ* = 0.834721, or 1669; the correct value is 1745. For the (3, 3, …, 3) vector, the net predicts *ϵ* = 0.673568, or 1347; the correct value is 1309.

**Fig 4:**
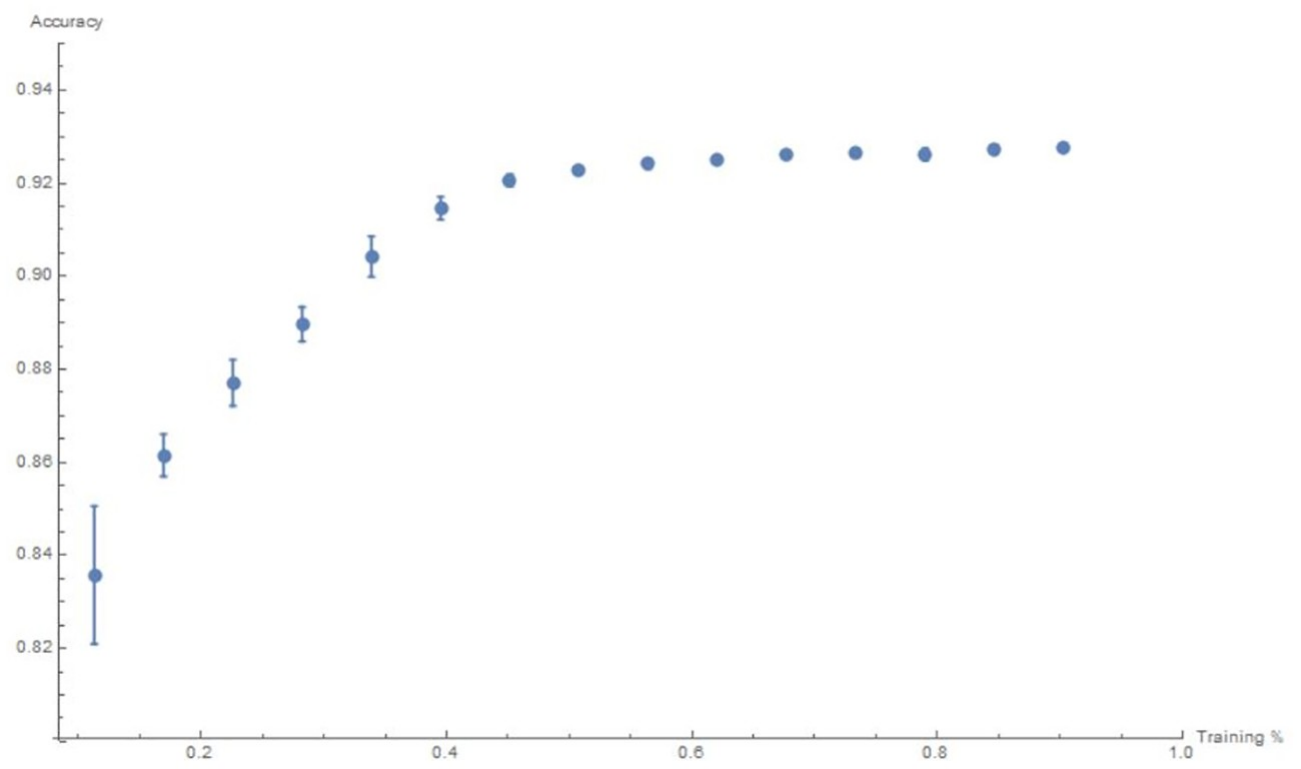
Learning curve for the R-square value versus fraction (seen) training data from 0 to 1.

We will use R-squared, a real number between 0 and 1, as a measure of accuracy of the machine-learning; the closer it is to 1, the better the fit (for a good reference on machine-learning and goodness of fit measure, cf. e.g. [25]). Our 30, 000 training set was only to illustrate the technique in detail. In general, we need to perform cross-validation by splitting the dataset. We split the data into a fraction x of random samples for training and validate on the complement 1 − x, done for training set from 30, 000 to 500, 000, in steps of 30, 000. The R-squared value is computed for each case as a measure of precision. Moreover, for each *x*, we repeat the random sampling 10 times, for which we get the error bars. The plot of the R-squared (with error bars) against the increase of percentage x of the size of the training set con-stitutes a learning curve which illustrates how the neural network responds to the data; this is shown in Fig 4.

As a comparison, one might imagine that since there is an underlying pattern being machine-learnt, a simple regression might suffice. That is, could one fit a hyperplane $f\left(x_{i}\right)=a_{0}+\sum_{i=1}^{12}a_{i}v_{i}$ to the data? We perform this over the entire dataset, and find that the best multi-linear regression obtains only *R*^2^ = 0.575428. Introducing non-linearity and more parameters, such as fitting $f\left(x_{i}\right)=a_{0}+\sum_{i=1}^{12}a_{i}v_{i}+\sum_{i=1}^{12}b_{i}v_{i}^{2}$ does not do much better, at *R*^2^ = 0.665484. The inherent complexity (non-linearity) of the problem is therefore best captured by our neural network approach.

## Supporting information

S1 File Mathematica notebook implementing the neural network described in the article. (NB)

S2 File Python Jupyter notebook implementing similar neural networks. (IPYNB)

## References

[1] Twarock R, Stockley PG. RNA-mediated virus assembly: mechanisms and consequences for viral evolution and therapy. Annual Review of Biophysics. 2019;. 10.1146/annurev-biophys-052118-115611 30951648 PMC7612295

[2] Dechant PP, Twarock R, et al. Models of viral capsid symmetry as a driver of discovery in virology and nanotechnology. The Biochemist. 2021;43(1):20–24. 10.1042/bio_2020_102

[3] Stockley PG, White SJ, Dykeman E, Manfield I, Rolfsson O, Patel N, et al. Bacteriophage MS2 genomic RNA encodes an assembly instruction manual for its capsid. Bacteriophage. 2016;6(1):e1157666. 10.1080/21597081.2016.1157666 27144089 PMC4836477

[4] Koning RI, Gomez-Blanco J, Akopjana I, Vargas J, Kazaks A, Tars K, et al. Asymmetric cryo-EM reconstruction of phage MS2 reveals genome structure in situ. Nature communications. 2016;7. 10.1038/ncomms12524 27561669 PMC5007439

[5] Patel N, Dykeman EC, Coutts RH, Lomonossoff GP, Rowlands DJ, Phillips SE, et al. Revealing the density of encoded functions in a viral RNA. Proceedings of the National Academy of Sciences. 2015;112(7):2227–2232. 10.1073/pnas.1420812112 25646435 PMC4343168

[6] Twarock R, Leonov G, Stockley PG. Hamiltonian path analysis of viral genomes. Nature communications. 2018;9(1):2021. 10.1038/s41467-018-03713-y 29789600 PMC5964074

[7] Patel N, Wroblewski E, Leonov G, Phillips SE, Tuma R, Twarock R, et al. Rewriting nature’s assembly manual for a ssRNA virus. Proceedings of the National Academy of Sciences. 2017; p. 201706951. 10.1073/pnas.1706951114 29087310 PMC5699041

[8] Zlotnick A. To build a virus capsid: an equilibrium model of the self assembly of polyhedral protein complexes. Journal of molecular biology. 1994;241(1):59–67. 10.1006/jmbi.1994.1473 8051707

[9] Bingham RJ, Dykeman EC, Twarock R. RNA Virus Evolution via a Quasispecies-Based Model Reveals a Drug Target with a High Barrier to Resistance. Viruses. 2017;9(11):347. 10.3390/v9110347 PMC5707554 29149077

[10] Gillespie DT. Exact stochastic simulation of coupled chemical reactions. The journal of physical chemistry. 1977;81(25):2340–2361. 10.1021/j100540a008

[11] Dykeman EC. A Model for Viral Assembly around an Explicit RNA Sequence Generates an Implicit Fitness Landscape. Biophysical Journal. 2017;113(3):506–516. 10.1016/j.bpj.2017.06.037 28793206 PMC5550301

[12] Singh AR, Košmrlj A, Bruinsma R. Finite temperature phase behavior of viral capsids as oriented particle shells. Physical review letters. 2020;124(15):158101. 10.1103/PhysRevLett.124.158101 32357054 PMC7219451

[13] Li S, Orland H, Zandi R. Self consistent field theory of virus assembly. Journal of Physics: Condensed Matter. 2018;30(14):144002. 10.1088/1361-648X/aab0c6 29460850 PMC7104907

[14] Twarock R, Bingham RJ, Dykeman EC, Stockley PG. A modelling paradigm for RNA virus assembly. Current Opinion in Virology. 2018;31:74–81. 10.1016/j.coviro.2018.07.003 30078702 PMC6281560

[15] Eigen M. Viruses: evolution, propagation, and defense. Nutrition reviews. 2000;58(s1). 10748612 10.1111/j.1753-4887.2000.tb07798.x

[16] Lauring AS, Andino R. Quasispecies theory and the behavior of RNA viruses. PLoS pathogens. 2010;6(7). 10.1371/journal.ppat.1001005 20661479 PMC2908548

[17] Lauring AS, Andino R. Exploring the fitness landscape of an RNA virus by using a universal barcode microarray. Journal of virology. 2011;85(8):3780–3791. 10.1128/JVI.02217-10 21289109 PMC3126141

[18] Degiacomi MT. Coupling Molecular Dynamics and Deep Learning to Mine Protein Conformational Space. Available at SSRN 3213915. 2018. 10.1016/j.str.2019.03.018 31031199

[19] Hastie T, Tibshirani R, Friedman J. The elements of statistical learning: data mining, inference, and prediction. Springer Science & Business Media; 2009.

[20] He YH. Deep-learning the landscape. arXiv preprint arXiv:170602714. 2017.

[21] He YH. Machine-learning the string landscape. Phys Lett. 2017;B774:564–568. 10.1016/j.physletb.2017.10.024

[22] He YH. The Calabi-Yau Landscape: from Geometry, to Physics, to Machine-Learning. to appear in Lecture Notes in Mathematics, Springer; 2021.

[23] Wolfram Research Inc. Mathematica, Version 11.3.

[24] Martín Abadi et al. TensorFlow: Large-Scale Machine Learning on Heterogeneous Systems;. Available from: http://tensorflow.org/.

[25] Haykin S. Neural Networks: A Comprehensive Foundation. 1st ed. Upper Saddle River, NJ, USA: Prentice Hall PTR; 1994.

